# 

**Published:** 1854-01

**Authors:** 


### RECEIPTS

### FOR THE JOURNAL DURING THE MONTH OF JANUARY.

ILLINOIS.

| | |
|---|---|
| Dr. B. Harris, | $3 00 |
| " R. L. Metcalf, | 2 00 |
| " F. Bry, | 6 00 |
| " J. Grinstea, | 2 00 |
| " C. Goodbrake, | 2 00 |
| " E. Friend, | 2 00 |
| " Cheney & Noble, | 4 00 |
| " F. D. Martin, | 3 00 |
| " Thos. D. Washburn, | 1 00 |
| " Hosea Davis, | 3 00 |
| " K. E. Rich, | 2 00 |
| " L. A. Hannaford, | 2 00 |
| " G. W. Minard, | 3 00 |
| " S. Y. Baldwin, | 2 00 |
| " Chas. G. Strohecker, | 2 00 |
| " Mitchell & Reagan, | 4 00 |
| " Geo. Ryon, | 2 00 |
| " A. A. Smith | 2 00 |
| " H. Nance, | 2 00 |
| " A. A. Smith, | 2 00 |

INDIANA.

| | |
|---|---|
| Dr. W. Lockhart, | $2 00 |
| " A Chapman, | 2 00 |
| " D. Newell, | 2 00 |
| " C. Palmerton, | 2 00 |
| " Chas. Bracket, | 2 00 |

WISCONSIN.

| | |
|---|---|
| Dr. H. D. Adams, | $2 00 |
| " J. L. Ingersell, | 2 00 |
| " R T. Hayes, | 2 00 |
| " A. Everhard, | 2 00 |
| " Thos. Secor, | 2 00 |
| " S. Ramsay, | 2 00 |

MICHIGAN.

| | |
|---|---|
| Dr. J. D. Woodworth, | $2 00 |
| " H. Lockwood, | 2 00 |
| " A. W. Mack, | 4 00 |
| " W. H. Fox, | 6 00 |

IOWA.

| | |
|---|---|
| Dr. F. H Cooledge, | $4 00 |
| " H. Clements, | 2 00 |
| " H. W Hart, | 2 00 |

OHIO.

| | |
|---|---|
| Dr. Isaac Hazlitt, | $2 00 |

MISSOURI,

| | |
|---|---|
| Dr. A. F. Barnett, | $2 00 |

PENNSYLNANIA.

| | |
|---|---|
| Dr. Samuel Strohecker, | 2 00 |

### PROSPECTUS OF VOLUME III.

### OF THE NEW SERIES OF THE

### NORTH-WESTERN
### Medical and Surgical Journal.

EDITED BY

W. B. HERRICK, M. D.,

*Professor of Anatomy and Physiology in Rush Medical College, one of the Surgeons of the Ill. General Hospital, etc. etc.*

AND

H. A. JOHNSON, A. M., M. D.,

*Lecturer on Physiology and Histology in Rush Medical College.*

THE THIRD Volume of the New Series of the N. W. Medical and Surgical Journal, will commence, in January, 1854. Each No. will contain 48 pages, closely printed in good clear type, thus presenting at the end of the year a volume of nearly 600 pages.

The editors will devote their time exclusively to the Journal, and endeavor, in addition to a full and complete Domestic Summary, to present numerous extracts from English, and translations from French and German Medical periodicals.

In addition to the Communications from Contributors, the Original Department will contain, from time to time, reports of Cases treated, and of Observations made, in the two Hospitals, now fully organized in Chicago.

Every alternate No. of the Journal will contain 16 pages of Prof. N. S Davis' excellent work on on the "Pathology of Fever."

### TERMS:

TWO DOLLARS PER ANNUM, IN ADVANCE.

*If not Paid in Advance, Three Dollars per Annum will invariably be charged.*

As an inducement to Physicians and others to act as Agents in extending the circulation of the Journal,

| | | | | |
|---|---|---|---|---|
| Three Copies will be furnished to new subscribers, for | | | | $5 00 |
| Five Copies | " | " | " | 8 00 |
| Seven Copies, | " | " | " | 10 00 |

### TERMS OF ADVERTISING:

| | | | |
|---|---|---|---|
| One Page, One Year. . | $20 00 | One Page, Single Insertion, | $5 00 |
| One-half Page, One Year, | 12 00 | One-half Page, single insertion, | 3 00 |

*Communications for insertion in the Journal to be addressed to the* "Editors of the N. W Medical and Surgical Journal, Chicago, Ill.," *and all communications connected with the business of the Journal to be addressed to* "Ballantyne & Co., Chicago, Ill."

